# Circulating Extracellular Vesicles in Subarachnoid Hemorrhage Patients: Characterization and Cellular Effects

**DOI:** 10.3390/ijms241914913

**Published:** 2023-10-05

**Authors:** Elena Grossini, Teresa Esposito, Michela Viretto, Sakthipriyan Venkatesan, Ilaria Licari, Daniela Surico, Francesco Della Corte, Luigi Castello, Stefania Bruno, Marco Quaglia, Cristoforo Comi, Vincenzo Cantaluppi, Rosanna Vaschetto

**Affiliations:** 1 Laboratory of Physiology, Department of Translational Medicine, Università del Piemonte Orientale, 28100 Novara, Italy; sakthipriyan.venkatesan@uniupo.it; 2 Anesthesia and Intensive Care, Department of Translational Medicine, Università del Piemonte Orientale, 28100 Novara, Italy; expoterry@gmail.com (T.E.); michela.viretto@gmail.com (M.V.); ilarialicari.82@gmail.com (I.L.); francesco.dellacorte@med.uniupo.it (F.D.C.); rosanna.vaschetto@med.uniupo.it (R.V.); 3 Maggiore della Carità Hospital, 28100 Novara, Italy; daniela.surico@med.uniupo.it (D.S.); vincenzo.cantaluppi@med.uniupo.it (V.C.); 4 Gynecology and Obstetrics, Department of Translational Medicine, Università del Piemonte Orientale, 28100 Novara, Italy; 5 Internal Medicine, Department of Translational Medicine, Università del Piemonte Orientale, 28100 Novara, Italy; luigi.castello@med.uniupo.it; 6 Azienda Ospedaliera SS. Antonio e Biagio e Cesare Arrigo, 15121 Alessandria, Italy; marco.quaglia@med.uniupo.it; 7 Laboratory of Translational Research, Department of Medical Sciences, University of Torino, 10126 Torino, Italy; stefania.bruno@unito.it; 8 Nephrology, Department of Translational Medicine, Università del Piemonte Orientale, 28100 Novara, Italy; 9 Neurology Unit, Department of Translational Medicine, Università del Piemonte Orientale, 28100 Novara, Italy; cristoforo.comi@med.uniupo.it; 10 Sant’Andrea Hospital, 00189 Vercelli, Italy

**Keywords:** subarachnoid hemorrhage, endothelial function, delayed cerebral ischemia, vasospasm, extracellular vesicles

## Abstract

Circulating extracellular vesicles (EVs) may play a pathophysiological role in the onset of complications of subarachnoid hemorrhage (SAH), potentially contributing to the development of vasospasm (VP). In this study, we aimed to characterize circulating EVs in SAH patients and examine their effects on endothelial and smooth muscle cells (SMCs). In a total of 18 SAH patients, 10 with VP (VP), 8 without VP (NVP), and 5 healthy controls (HC), clinical variables were recorded at different time points. EVs isolated from plasma samples were characterized and used to stimulate human vascular endothelial cells (HUVECs) and SMCs. We found that EVs from SAH patients expressed markers of T-lymphocytes and platelets and had a larger size and a higher concentration compared to those from HC. Moreover, EVs from VP patients reduced cell viability and mitochondrial membrane potential in HUVECs and increased oxidants and nitric oxide (NO) release. Furthermore, EVs from SAH patients increased intracellular calcium levels in SMCs. Altogether, our findings reveal an altered pattern of circulating EVs in SAH patients, suggesting their pathogenic role in promoting endothelial damage and enhancing smooth muscle reactivity. These results have significant implications for the use of EVs as potential diagnostic/prognostic markers and therapeutic tools in SAH management.

## 1. Introduction

Aneurysmal subarachnoid hemorrhage (SAH) remains a significant global health challenge, characterized by high fatality and permanent disability rates [1,2], particularly among younger patients [3,4]. Accounting for 5% of all strokes, SAH is frequently associated with the spontaneous rupture of an aneurysm [2,5]. Even though its incidence has decreased over the past years due to lifestyle modifications and the adoption of public health measures, the occurrence of poor functional outcomes following SAH remains high [1,2].

Early brain injury (EBI), defined as cerebral damage occurring within 72 h after SAH, along with delayed cerebral ischemia (DCI), which refers to injury developing in the days to weeks following the hemorrhage, are major determinants of unfavorable neurological outcomes associated with SAH [3,6,7,8]. Although there is a better understanding of the pathogenesis of EBI, which involves a sudden increase in intracranial pressure followed by a decrease in cerebral blood flow due to hemorrhage, the mechanisms underlying DBI are less clear [9,10]. Previous studies have shown how a variety of different pathological processes, such as vasospasm (VP), oxidative damage, blood–brain barrier disruption, and inflammation [6,7,8,11,12] play a role in DBI occurrence. However, the significance and interplay of each of these pathways are yet to be fully elucidated.

In the context of SAH, a promising research field has recently emerged related the extracellular vesicles (EVs) [13]. Initially described as pro-coagulant “dust” surrounding activated platelets [14], EVs have been shown to be important mediators of physiological and pathological processes in various diseases. Indeed, they originate from both blood (i.e., platelets, leucocytes, and erythrocytes) and vascular cells (i.e., endothelial cells (ECs) and smooth muscle cells (SMCs)), and their regulatory and paracrine activities have been confirmed by numerous studies [15,16].

The role of circulating EVs in ischemic and hemorrhagic stroke has emerged as an intriguing area of research, as their levels have been found to correlate with clinical severity, extent of infarct area, and poor outcomes [17,18]. Following SAH, an increase in different subtypes of EVs has been demonstrated on the day of the hemorrhage, with variations in temporal profiles depending on the EV subtype [19,20]. Furthermore, elevated levels of endothelial-derived EVs correlate with an increased incidence of VP after SAH, while platelet-derived EVs have been found to play a role in DCI development [20].

Even though there are promising results suggesting the potential role of EVs as biomarkers for complications in SAH patients and their potential involvement in the pathophysiology of DCI, the existing data in this field are limited and findings often lack consistency among different studies. In particular, there are currently no data relating to both the characterization of circulating EVs in SAH patients and to their effects on ECs and SMCs, which are widely involved in the pathophysiology of SAH [21]. Therefore, the main objective of this study was to thoroughly characterize circulating EVs in SAH patients with and without VP, in terms of size, concentration, surface markers, and temporal changes and to examine their effects on vascular ECs and SMCs.

## 2. Results

### 2.1. Patients and Setting

Eighteen patients with severe SAH following the rupture of cerebral aneurysm were included in the present study. In addition, five age- and sex-matched healthy controls (HC) (three women and two men) aged 53 (46.5–61.5) years, were recruited at the same hospital. None of them suffered from major comorbidities, whereas 33% of SAH patients had hypertension, and 5% suffered from cardiovascular, respiratory, or chronic kidney disease (Table 1). VP, defined as the mean flow velocity greater than 120 cm/s in the middle cerebral artery measured by TCD, occurred in 8 patients, while the remaining 10 NVP subjects were not affected. Patients’ demographics and clinical characteristics are shown in Table 2. Patients (8 males, 10 females) had a median age of 54.5 years (IQR: 71–47.5) and aneurysm sites distributed evenly among the anterior, middle, and posterior circulation. In total, 5 patients underwent surgical clipping of the aneurysm, while the remaining 13 were subjected to endovascular coiling. Overall, the median GCS on ICU admission was 7 (IQR: 9–6), whereas the mean scores for the WFNS scale, Fisher scale, and HHS were 4.5 (IQR: 5–4), 3.5 (IQR: 4–3), and 4 (IQR: 5–3), respectively. The average length of stay at the ICU and hospital was 21 (IQR: 33.5–11) and 29 (IQR: 46.3–14) days, respectively. While age was significantly lower in VP vs. NVP patients (49.5 years, IQR: 43.5–52.8 vs. 70.5 years, IQR: 57.8–73.5; *p* < 0.05), no other significant differences were observed between the two groups. Regarding the neurological outcome, both patient populations exhibited predominantly poor outcomes, as evidenced by the assessments performed using the mRS and GOS-E scales at 3 and 6 months after SAH.

### 2.2. Characterization of Circulating EVs

**Figure 1:**
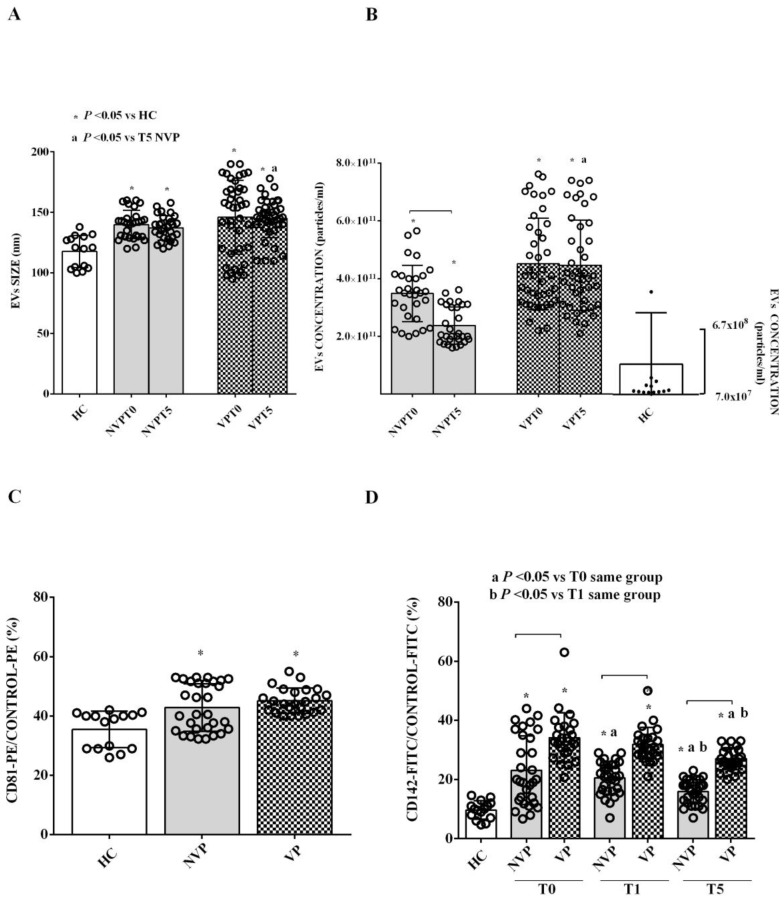
NanoSight analysis of extracellular vesicle (EV) size (**A**) and concentration (**B**) and expression levels of CD81 (**C**) and CD142 (**D**). EVs were isolated from patients with vasospasm (VP), without vasospasm (NVP), or healthy controls (HCs). The results are expressed as the mean ± SD of three different measurements. CD81 and CD142 are expressed in relation to the PE or FITC control. Square brackets indicate significance between groups (*p* < 0.05). T0: ICU admission; T1: 24 h after ICU admission; T5: seven days after bleeding.

NanoSight analysis of EVs revealed a marked increase in both their size and concentration in SAH patients compared to HC (Figure 1). While there were no significant EV size differences between VP and NVP patients at T0, we did observe a significantly larger size of EVs derived from VP patients (VP-EVs) at T5 compared to that of their NVP counterparts (NVP-EVs) (Figure 1A). When we measured the EV concentration, it was found to be higher at T0 compared to T5 only in NVP patients. At T5, the concentration of VP-EVs was higher than that of NVP-EVs (Figure 1B). Analysis of CD81, which was expressed in the EVs from both SAH patients and HC, confirmed the exosomal origin of the EVs. Notably, CD81 expression was higher in VP patients than in HC (Figure 1C). In addition, both VP- and NVP-EVs showed a more robust expression of CD142, also known as tissue factor (TF), compared to HC (Figure 1D). Although a decreasing trend was observed in VP and NVP patients over time, at T5, TF was still more highly expressed in the EVs of patients than in HC. Finally, TF expression in VP patients was consistently higher than that observed in NVP patients across all time points (Figure 1D).

**Figure 2:**
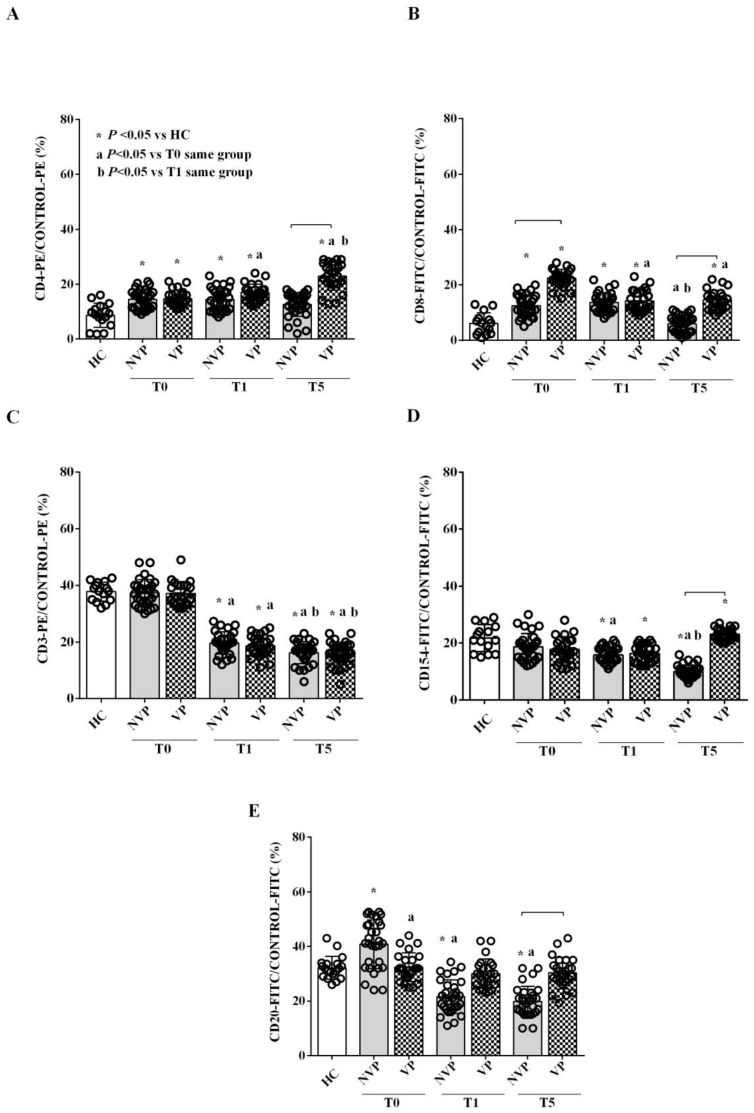
Expression of lymphocyte T (CD4, CD8, CD3, CD154; (**A**–**D**)) and lymphocyte B (CD20; (**E**)) markers in VP-, NVP-, and HC-EVs. The results are expressed as the mean ± SD of at three different measurements. All markers are expressed in relation to the PE or FITC control. Square brackets indicate significance between groups (*p* < 0.05). The abbreviations and layout are the same as those described in the legend to Figure 1.

As shown in Figure 2A,B, both VP-and NVP-EVs displayed higher expression levels of the T-lymphocyte markers CD4 and CD8 at T0 and T1 compared to HC. At T5, only VP patients maintained elevated expression levels of these markers compared to HC. Regarding CD3 and CD154, no differences were observed between patients and controls at T0, whereas a reduction in CD3 expression was observed at T1 and T5 in all patients. Notably, at T5, CD154 expression in VP-EVs was higher than that observed in NVP- and HC-EVs (Figure 2C,D). In contrast, NVP-EVs showed a higher expression of CD20, a B-lymphocyte marker, at T0 compared to VP- and HC-EVs. This expression gradually decreased at T1 and T5, reaching lower levels than those observed in HC. No changes were detected in CD20 expression in VP-EVs over time (Figure 2E).

**Figure 3:**
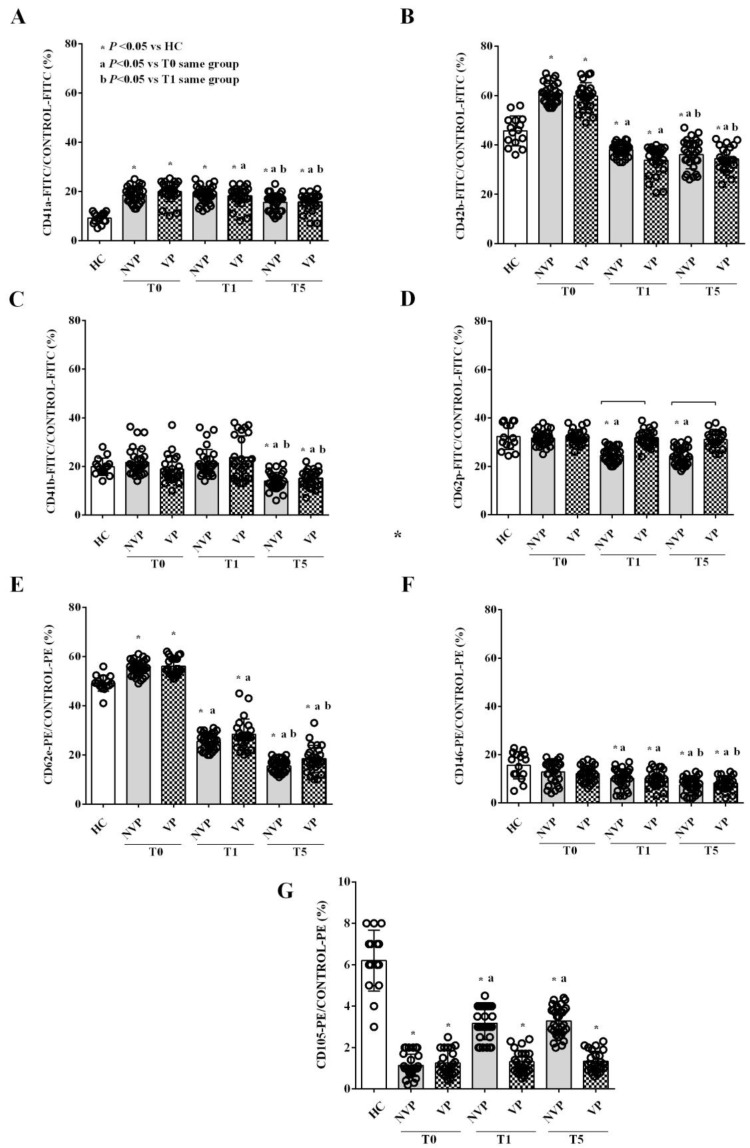
Expression of platelet (**A**–**D**) and endothelial (**E**–**G**) markers in VP-, NVP-, and HC-EVs. The results are expressed as the mean ± SD of at least three different measurements. All markers are expressed in relation to the PE or FITC control. Square brackets indicate significance between groups (*p* < 0.05). The abbreviations and layout are the same as those described in the legend to Figure 1.

Both VP- and NVP-EVs showed higher expression levels of CD41a and CD42b at T0 compared to HC-EVs. At subsequent time points, while CD41a expression remained high, that of CD42b decreased (Figure 3A,B). As for other platelet markers, we observed no changes at T0 for both CD41b and CD62p, whereas CD62p and CD41b expression levels were downregulated in NVP-EVs at T1 and T5 and in both VP- and NVP-EVs at T5, respectively (Figure 3C,D). Interestingly, both VP- and NVP-EVs showed decreased expression of CD62e and CD146, two endothelial markers of adhesion, at T1, and even more so at T5 (Figure 3E,F). In terms of CD105, a reduced expression was observed in VP- and NVP-EVs compared to HC-EVs. Furthermore, while CD105 increased in NVP-EVs over time, it remained unchanged in VP-EVs (Figure 3G).

From T0 to T5, the EVs characterized through FACS amounted to about 75–80% of the total EVs in both the VP and NVP patients.

In Table 3, the percentage of each type of EV among the total EV populations is reported.

### 2.3. In Vitro Studies

**Figure 4:**
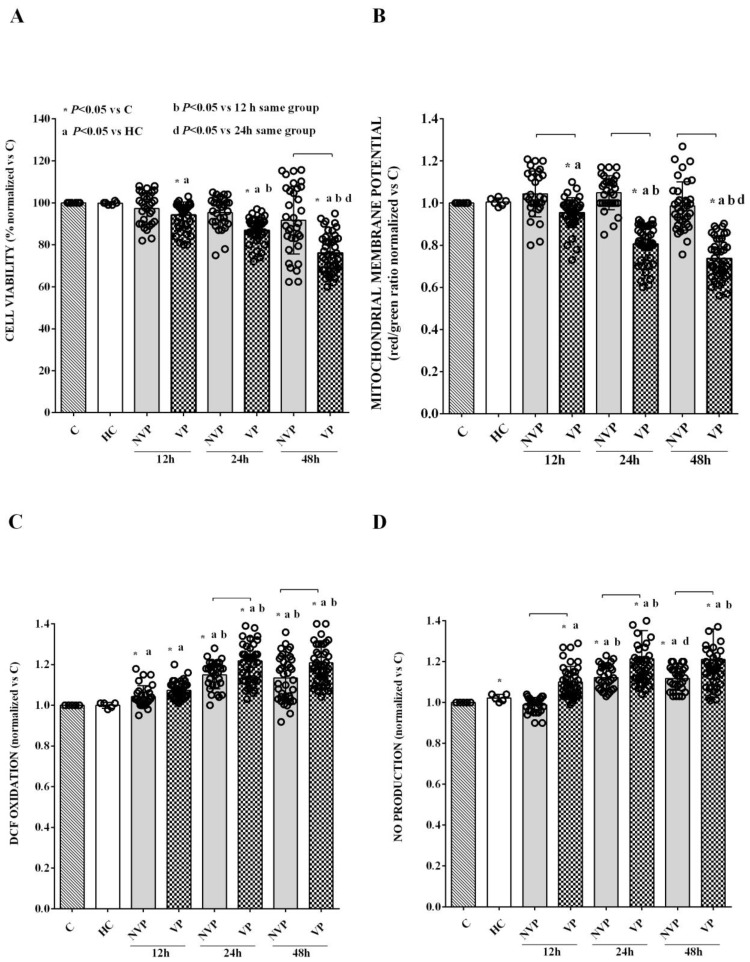
EVs were isolated from patients and HCs at T0 and used to stimulate HUVECs (50,000 EVs/cell) for 12, 24, and 48 h. The time-course effects on cell viability (**A**), mitochondrial membrane potential (**B**), reactive oxygen species (ROS) release (measured as DCF oxidation) (**C**), and nitric oxide (measured as nitrite production) (**D**) are shown. The results are expressed as the mean ± SD of experiments performed in triplicate. Square brackets indicate significance between groups (*p* < 0.05). C: untreated cells; DCF: 2,7-dichlorodihydrofluorescein. The other abbreviations are the same as those described in the legend to Figure 1.

In vitro experiments were conducted to assess the effects of VP- vs. NVP-EVs on HUVECs. Stimulation of HUVECs with VP- EVs isolated at T0 resulted in a significant reduction in cell viability and mitochondrial membrane potential, as well as an increase in ROS and NO release after 12 h of exposure. These effects were further amplified at 24 h and 48 h of stimulation (Figure 4). On the other hand, no significant changes were observed in cell viability and mitochondrial membrane potential when HUVECs were stimulated with NVP-EVs (Figure 4A, B). By contrast, an increase in ROS release was observed as early as 12 h of stimulation, which further increased at 24 h (Figure 4C). These elevated levels of ROS release remained stable during the 48h stimulation period. Conversely, an increase in nitric oxide (NO) release was only observed at 24 and 48h of stimulation (Figure 4D). It is important to note that the effects of NVP-EVs on ROS and NO release at T1 and T5 were significantly reduced compared to those elicited by VP-EVs at the same time points.

**Figure 5:**
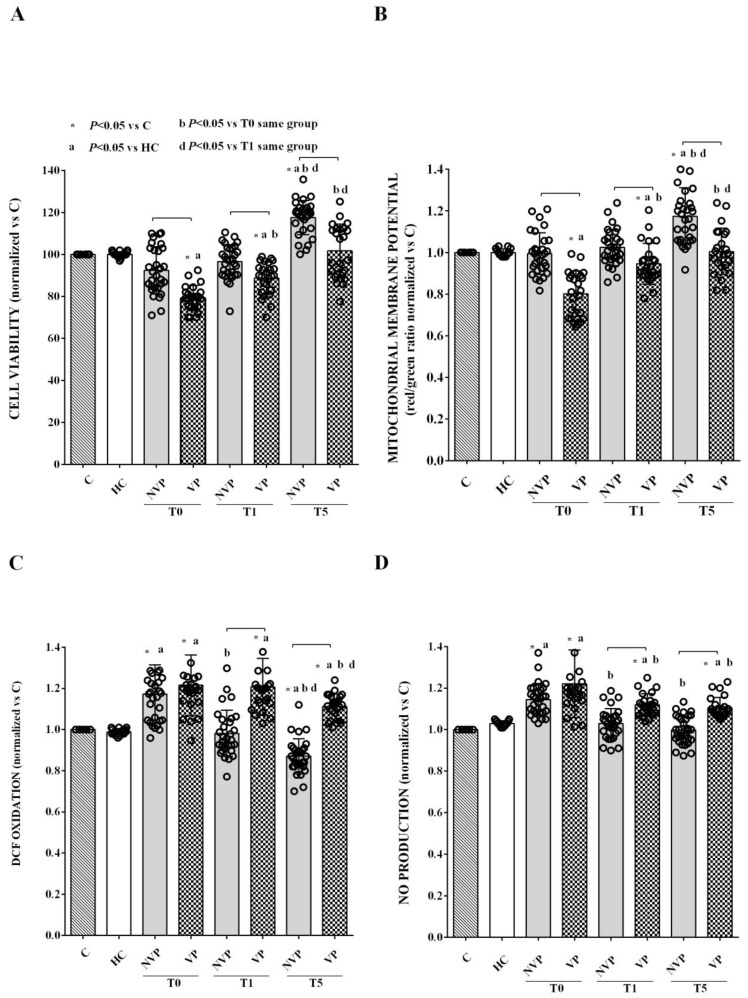
Effects of 48 h stimulation of HUVECs with VP-, NVP-, or HC-EVs (50,000 EVs/cell) on cell viability (**A**), mitochondrial membrane potential (**B**), reactive oxygen species release (measured as DCF oxidation) (**C**), and nitric oxide (measured as nitrite production) (**D**). The results are expressed as the mean ± SD of experiments performed in triplicate and repeated at least three times. Square brackets indicate significance between groups (*p* < 0.05). The other abbreviations are the same as those described in the legend to Figure 1.

Based on the results obtained with the time-course experiments, we selected the 48 h stimulation period for subsequent experiments in HUVECs, which were treated with the EVs isolated at T1 and T5. The results showed VP-EVs at both T1 and T5 reduced cell viability and mitochondrial membrane potential up to T1 compared to HC-EVs and untreated cells (Figure 5A,B). In contrast, VP-EVs continued to induce a more pronounced release of ROS and NO from HUVECs up to T5 compared to HC-EVs and untreated cells (Figure 5C,D).

As for NVP-EVs, we found some differences in their effects on HUVECs compared to VP-EVs. NVP-EVs isolated at T0 and T1 did not show any significant effect on cell viability and mitochondrial membrane potential, while at T5, they enhanced these variables (Figure 5A,B). Regarding ROS and NO release, only NVP-EVs isolated at T0 induced an increase, whereas those isolated at the other time points did not significantly affect these variables or even decreased their values, as in the case of ROS release (Figure 5C,D). Moreover, it is important to point out that, for most time points and assays (excluding ROS and NO at T0), the effects triggered by VP-EVs were more pronounced compared to those induced by NVP-EVs.

Overall, the results obtained on HUVECs have highlighted how the EVs from SAH patients were able to cause damages to mitochondrial function with an increase in the release of ROS and NO. Furthermore, we found differences between VP and NVP patients. Given that endothelial and smooth muscle cells represent a network capable of interacting with each other, and are implicated in the pathophysiology of SAH through changes of the contraction capacity of smooth muscle cells, we, therefore, wanted to examine whether the EVs could influence calcium movements in the smooth muscle cells, as a direct action.

**Figure 6:**
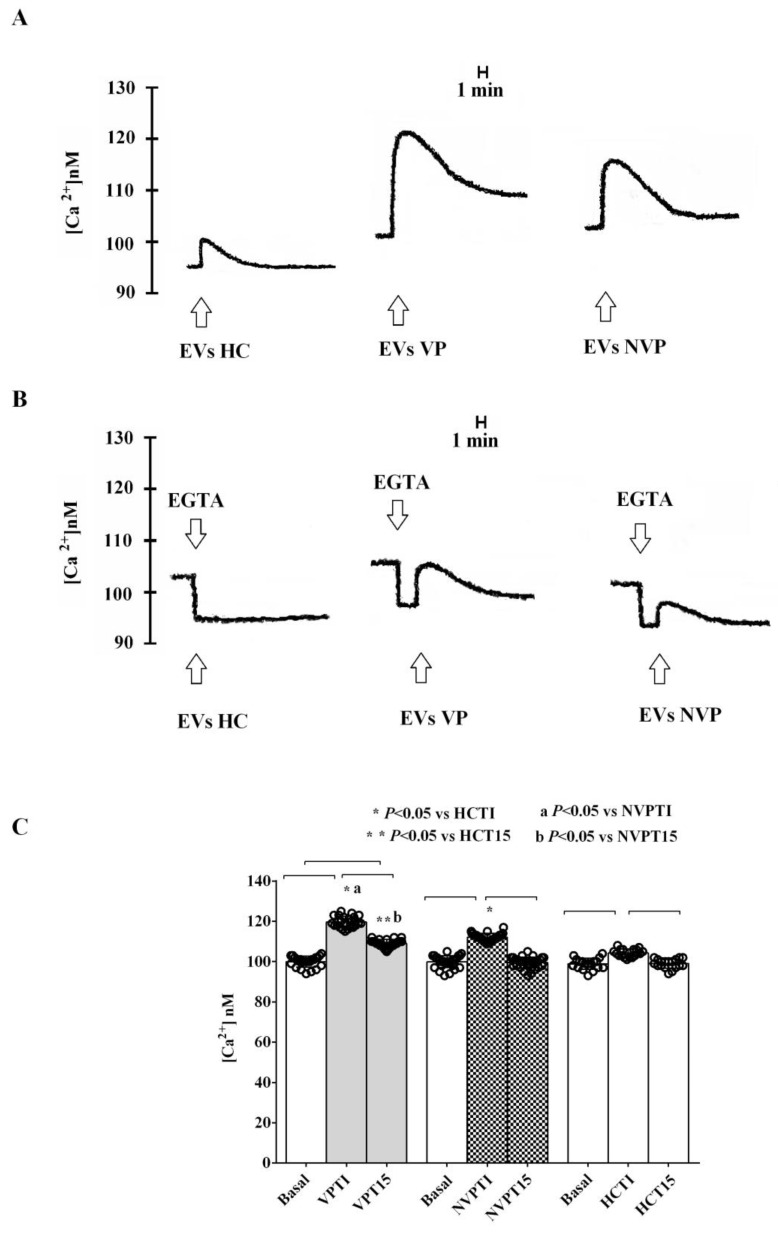
Effects of VP-, NVP-, or HC-EVs (T0) stimulation (50,000 EVs/cell) of C2C12 cells on calcium mobilization. (**A**,**B**) Representative examples of experiments performed in triplicate, are shown. (**C**) Results obtained in repeated experiments expressed as mean ± SD. Basal: baseline intracellular calcium levels (in C2C12 stimulated with PBS only). Square brackets indicate significance between groups (*p* < 0.05). TI: measurements of intracellular calcium immediately after the EVs administration; T15: measurements of intracellular calcium 15 min after the EVs administration. The other abbreviations are the same as those described in the legend to Figure 1.

**Figure 7:**
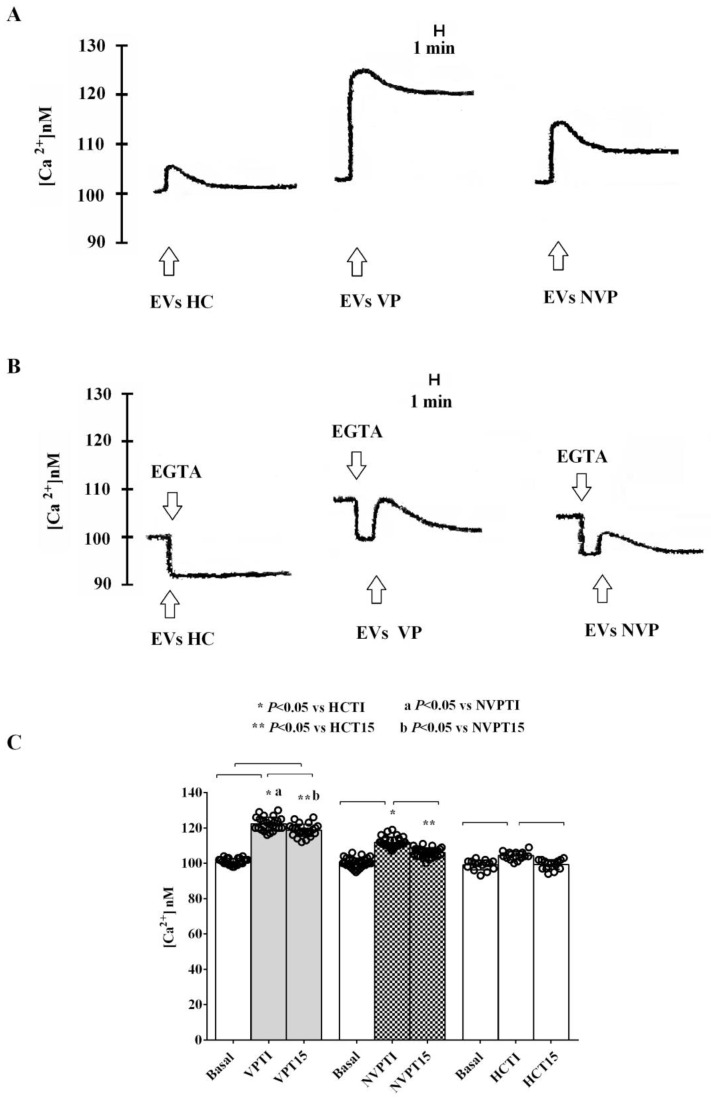
Effects of VP-, NVP-, or HC-EVs (T1) stimulation (50,000 EVs/cell) of C2C12 cells on calcium mobilization. (**A**,**B**) Representative examples of experiments performed in triplicate, are shown. (**C**) Results obtained in experiments performed in triplicate and repeated at least three times are expressed as mean ± SD. Basal: baseline intracellular calcium levels. Square brackets indicate significance between groups (*p* < 0.05). The other abbreviations are the same as those described in the legend to Figure 1.

**Figure 8:**
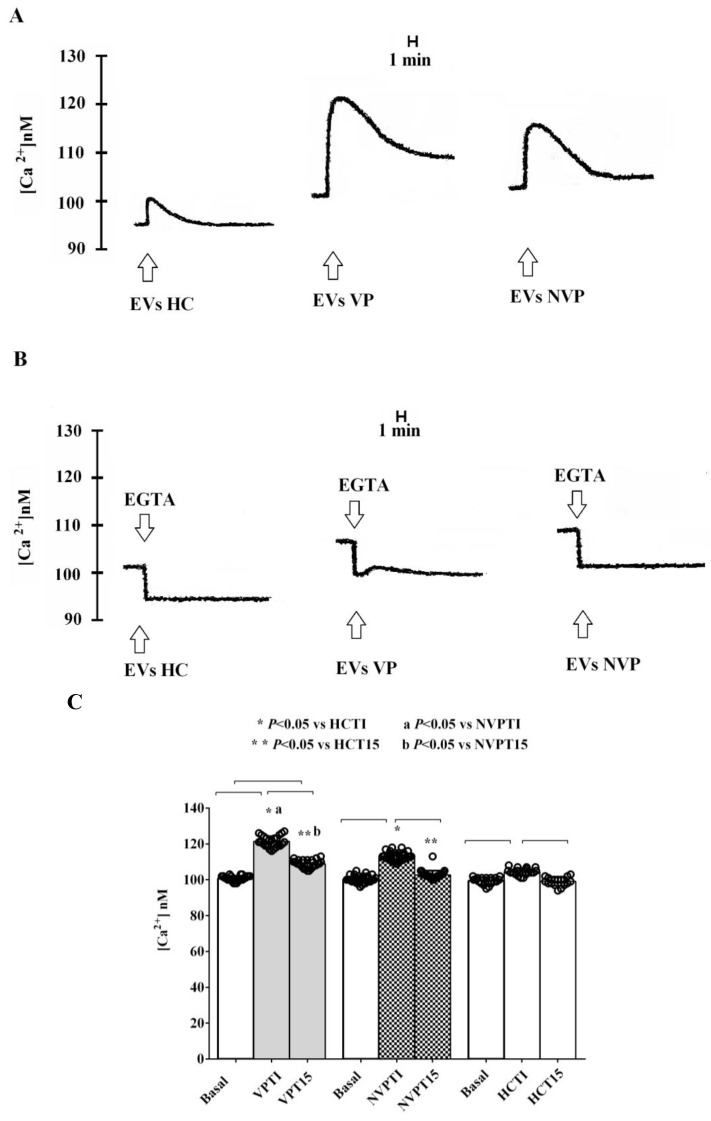
Effects of VP-, NVP-, and HC-EVs (T5) stimulation (50,000 EVs/cell) of C2C12 cells on calcium mobilization. (**A**,**B**) Representative examples of experiments performed in triplicate, are shown. (**C**) Results obtained in experiments performed in triplicate and repeated at least three times are expressed as mean ± SD. Basal: baseline intracellular calcium levels. EVs: extracellular vesicles. Square brackets indicate significance between groups (*p* < 0.05). The layout and other abbreviations are the same as those described in the legend to Figure 1.

Thus, we stimulated C2C12 cells for 48 h with VP-, NVP-, or HC-EVs isolated at T0. As shown in Figure 6A,C, and in Table 4, the administration of either VP- or NVP-EVs immediately increased intracellular calcium levels, far exceeding the effect observed with HC-EVs-treated cells. Furthermore, the effects were more pronounced in VP-EVs- than NVP-EVs-treated cells. It is noteworthy that even after 15 min of VP-EVs administration, the intracellular calcium levels remained higher than those observed at baseline. Conversely, by stimulating cells with either NVP- or HC-EVs, intracellular calcium levels returned to baseline values. In addition, EVs isolated at T1 exhibited a similar effect on intracellular calcium to that observed at T0, but with significantly higher levels at 15 min after EVs administration, reaching a plateau, particularly with VP-EVs (Figure 7A,C). Furthermore, the effect of EVs on intracellular calcium levels persisted even when we stimulated C2C12 cells with VP-or NVP-EVs at T5. Figure 8A,C, and Table 4, which demonstrates that, once again, VP-EVs induced the highest increase in calcium levels, both immediately and after 15 min of exposure. Fittingly, as depicted in Figure 6, Figure 7 and Figure 8B, and Table 4, the presence of EGTA significantly diminished all the effects of EVs. Specifically, the EVs isolated at T5 failed to elicit a significant increase in intracellular calcium in EGTA-treated C2C12 cells (Figure 8B). These findings indicate that the calcium mobilized by EVs is partly derived from the extracellular environment.

## 3. Discussion

The results of this study provide evidence for an altered profile of circulating EVs in the plasma of patients with SAH. We show that these EVs have the ability to induce damage in vascular ECs and disrupt calcium mobilization in SMCs. Furthermore, differences were observed in the characterization of EVs and their effects on cells between VP and NVP, even between 24 h (T0) and 7 days (T5) after the bleeding event.

Despite recent advances in diagnostic, neurosurgical, and anesthetic techniques, as well as preoperative and postoperative patient management, only a small proportion of SAH survivors achieve favorable neurological outcomes without a decline in QoL [22]. The outcome following aneurysmal SAH is influenced by various factors, including the occurrence of complications such as VP and delayed cerebral ischemia (DCI), which can lead to varying degrees of neurological impairment and, in severe cases, even mortality [5,6,7,8,23]. Cerebral VP, in particular, has been described as the leading cause of morbidity and mortality in SAH patients [24,25,26,27]. Thus, increasing our knowledge about both EBI and DCI pathophysiology is essential for the improvement of patient clinical outcome, especially since SAH treatment is associated with several complications [28,29]. Furthermore, the diagnosis of SAH is currently limited by the lack of accessible molecular biomarkers that reflect the underlying disease processes [30]. It is therefore crucial to gain a better understanding of the pathophysiological changes that occur in the early phase after SAH, which include microvascular filling defects, endothelial dysfunction, inflammation, and microarterial constriction [31,32,33,34,35]. Among the various mediators of damage found in SAH, extracellular vesicles (EVs) have emerged as potential mediators of secondary pathogenesis, particularly in relation to VP. Previous studies have demonstrated that EVs can cross the blood–brain barrier and facilitate the transfer of proteins, lipids, mRNAs, and miRNAs, ultimately leading to protein expression in target cells [20]. Furthermore, changes in EV-related miRNA have been reported to be involved in the development of cerebral VP after SAH through the induction of SMC proliferation and changes in endothelial tissue functions [36]. However, despite the existing data on the role of EVs in SAH and SAH-related complications, our knowledge in this field remains limited and further investigation is needed. Therefore, in this study, we aimed to isolate EVs from the plasma of HC and SAH patients (with or without VP) at different time points (T0, T1, and T5) and characterize them in terms of size, concentration, and cellular origin.

It should be noted that those issues have already been examined by previous studies. In most of them, the EVs were mostly characterized for main platelet/leukocytes/erythrocytes markers expression and concentration and the data were related with certain clinical issues [19,20,37,38]. In the study by Amabile et al. [38], the effects of EVs on rat aortic rings were evaluated in terms of relaxation and cyclic guanosine monophosphate levels as well.

In our study, we combined the characterization of circulating EVs in SAH patients at various times with the cytopathic effect on two cell lines, endothelial cells and smooth muscle cells, which are considered to play a key role in the pathophysiology of SAH [21].

The results obtained with NanoSight show that both the size and concentration of EVs from SAH patients are higher than those observed in EVs from HC. Interestingly, while there were no differences in size and concentration of EVs between VP and NVP patients at T0, these parameters were significantly increased in the VP group at T5. Furthermore, when comparing the two groups of patients, we observed a decrease in EVs concentration at T5 compared to T0 only in NVP patients. These findings are partly in agreement with previous literature, which has reported an increase in plasma EVs concentration in SAH patients on the day of bleeding [19,20]. However, we did not detect a decrease in EVs concentration in all patients but rather specifically in NVP. Additionally, contrary to previous reports [39,40], we found no differences in EVs parameters between VP and NVP patients at T0, but only at T5. Thus, altogether, these results emphasize the complexity of EVs dynamics in SAH and highlight the need for further exploration to fully understand their role in the pathophysiology of the disease and its associated complications.

It should also be noted that the size of the EVs from SAH patients was larger than that of HC-EVs across all time points. Furthermore, VP-EVs at T5 displayed a larger size than that of NVP-EVs. Although limited knowledge exists about this topic, our observations align with previous findings in patients with traumatic brain injury, where EVs exhibited an increased size at 4–7 days post-injury compared to controls [41]. In addition, the EVs isolated from patient plasma at T0 showed a higher expression of some lymphocyte/platelet markers compared to HC. Similar results were obtained for CD62e (E-selectin), which is considered a marker of activated ECs and mediates leukocyte rolling [42], whereas the expression of CD105 (endoglin), usually seen as an optimal indicator of the proliferation of human ECs [43], was reduced. At T1, the EVs of patients demonstrated an increased expression of CD4, CD8, and CD41a compared to HC.

If we consider the temporal pattern, we observed that CD4 and CD8 expression increased in VP-EVs, while a decrease was observed in NVPs compared to HC. CD154, a member of the TNF superfamily of molecules primarily expressed on activated T cells [44], was downregulated in NVP patients and upregulated at T5 in VP ones. The expression of CD20 was solely reduced in NVP-EVs, while that of CD3 was reduced in both groups. Regarding platelet markers, CD41a remained elevated in the EVs from both VP and NVP patients, while CD42b decreased in both groups, and CD62p decreased exclusively in NVP patients. The evaluation of the endothelial markers CD62e, CD105, and CD146 revealed a reduced expression at T1 and T5 in both patient groups. Notably, the expression of CD142, also known as TF, was increased in patient-derived EVs compared to HC at T0, with a more marked progressive reduction over time in NVP patients. Therefore, our data indicate a persistent pattern of inflammatory and platelet-derived EVs, particularly in VP patients, one week after the bleeding event. In addition, the analysis of the EV-associated endothelial markers showed a reduction in those involved in endothelial junction and angiogenesis [45], suggesting endothelial dysfunction and a loss of endothelial integrity a few days after the bleeding event [46,47]. Lastly, we found increased expression of TF at T5 in the EVs from SAH patients, especially in the VP group. Since TF is involved in the activation of the coagulation cascade, our findings suggest the persistence of an altered coagulation homeostasis related to endothelial dysfunction [19].

Having found the aforementioned alterations in SAH-patient circulating EVs patterns, we sought to determine their potential harmful effects on cell lines of endothelial origin, such as HUVECs, and on smooth muscle cells, such as C2C12. We chose these two cell lines as they represent the major components of the vascular wall and are implicated in the pathophysiology of SAH, also through alteration of the blood–brain barrier (BBB) [21]. The need to carry out in vitro experiments was related to the aim to identify a specific profile of circulating EVs in SAH patients, deriving from both the EVs characterization and the cellular effects. In order to achieve this objective, we analyzed the effects of the EVs in the in vitro model, represented by HUVECs and smooth muscle cells.

Our results show that HUVEC stimulation with VP-EVs vs. HC-EVs at T0 reduces cell viability and mitochondrial membrane potential, whereas it increases ROS and NO release as early as 12 h after stimulation. In NVP patients, we could only observe an increased ROS release at the same time point. Over subsequent time points, the harmful effects on HUVECs increased in both patient groups. Furthermore, in comparison with HC-EVs, stimulation of HUVECs for 48 h with VP-EVs at T0–T5 resulted in decreased cell viability and mitochondrial membrane potential up to T1, and increased release of ROS and NO, albeit with a decreasing trend from T0 to T5. Conversely, NVP-EVs only increased ROS and NO release at T0, while cell viability and mitochondrial membrane potential remained unchanged and even increased at T5. Hence, our findings in HUVECs show a potential harmful effect of EVs from SAH patients on ECs, suggesting their significant role in the onset of SAH-related complications, as also previously proposed [36]. Furthermore, our findings regarding mitochondrial dysfunction and increased oxidative stress have implications for further understanding the pathophysiology of SAH. Mitochondrial dysfunction, which can lead to the loss of mitochondrial membrane potential and increased ROS, is in fact considered a novel mechanism of EBI related to DCI, as well as post-SAH outcomes [48]. Consequently, mitochondria have emerged as potential therapeutic targets for SAH management. In this context, our data may provide valuable insights for the development of new therapeutic approaches in SAH patients.

The increase in NO release observed at all time points in HUVECs stimulated with the EVs from SAH patients, especially the VP group, implies that endothelial activation is characterized by oxidative and inflammatory processes. It is well known that NO can exert both protective and toxic effects, depending on its release levels and the specific isoforms of nitric oxide synthase (NOS) involved [49]. Previous studies have shown upregulation of neuronal (nNOS) and inducible (iNOS) NOS subtypes in SAH, resulting in elevated NO levels in the days following the bleeding event [11,50,51]. Excessive production of NO and its transformation into peroxynitrites may contribute to vasoconstriction rather than vasodilation [49], potentially contributing to the observed vasospasm in our patient group.

To further investigate the role of patient-derived EVs in VP development, we investigated their effects on the intracellular calcium levels in SMCs. The results obtained showed that, in both patient groups, calcium increased immediately after the administration of the EVs isolated at T0, and its release increased with T1 EVs, reaching a plateau. Furthermore, the effects were more pronounced and persistent in VP than NVP patients.

These data showing a more marked increase in cytosolic calcium in the presence of EVs isolated from patients with VP would account not only for the vasospasm observed in these patients, but also for their effects on HUVECs. The greater and more persistent increase in NO release we found in VP patients could, in fact, be related to a greater stimulation of the calcium-dependent variants of NOS, i.e., the endothelial NOS (eNOS) and nNOS [52,53]. Also, the higher and more persistent effect on ROS release by HUVECs treated with the EVs of VP patients could be related to the greater increase in cytosolic calcium in C2C12 [54,55,56]. In this way, changes in the network between vascular endothelial cells and smooth muscle cells could be involved in the genesis of the vascular effects induced by the EVs of SAH patients with VP. Since those cell lines are components of BBB, their injury could be accompanied by alterations of the BBB, which occur immediately after SAH and are involved in the development of early brain injury, as well as delayed cerebral ischemia.

## 4. Materials and Methods

### 4.1. Patients

Patients were recruited from October 2017 to January 2020 at the general Intensive Care Unit (ICU) of the university hospital “Maggiore della Carità” in Novara, Italy. Clinical data from a portion of this patient population have been previously published [57]. The approval of the Ethics Committee (protocol CE 115/17) was obtained, and all participants provided written informed consent, either personally if capable, or through a legal guardian when necessary. The study was performed in accordance with the ethical standards outlined in the 1964 Declaration of Helsinki and its subsequent amendments or comparable ethical standards.

Inclusion criteria for the study were as follows: (1) age above 18 years; (2) diagnosis of subarachnoid hemorrhage (SAH) from a ruptured aneurysm confirmed by CT scan or angiography within 24 h; and (3) planned placement of an external ventricular or spinal drainage catheter. Exclusion criteria included an age below 18 or above 80 years, bleeding occurring more than 24 h before inclusion, and the presence of coagulopathy or ongoing anticoagulant/antiplatelet therapy.

Demographic characteristics, coexisting comorbidities, symptoms at clinical presentation, and radiological findings performed at hospital entrance were collected. The Hunt and Hess scale (HHS), Fisher Scale, World Federation of Neurosurgical Societies (WFNS) scale, and Glasgow Coma Scale (GCS) scores were also recorded on admission [58,59,60,61]. Patients admitted to the ICU were treated according to current clinical practice. Transcranial doppler ultrasound (TCD) was used on the first day to establish a baseline velocity of cerebral flow in the major intracranial blood vessels and subsequently performed daily starting from Day 4 after ICU admission. Angiography with endovascular nimodipine infusion was considered if patients exhibited clinical worsening or increased Doppler velocity. VP was classified as mild when the mean velocity in the middle cerebral artery reached 120–150 cm/s, moderate if the velocity was 150–200 cm/s, and severe if it exceeded 200 cm/s. The neurological outcome of the patients was assessed at 3–6 months after hospital discharge using the Glasgow Outcome Scale Extended (GOS-E) and the modified Rankin Scale (mRS) [62,63]. In addition, five age- and sex-matched healthy controls (HC) were recruited at the same hospital.

### 4.2. Blood Sample Collection

In patients and HC, blood samples were taken using BD Vacutainer tubes (sodium heparin as anticoagulant). Plasma samples were collected at T0 (ICU admission, within 24 h after SAH occurrence), T1 (24 h after ICU admission), and T5 (7 days following the bleeding). After centrifugation for 10 min at 3100 rpm and 4 °C with a centrifuge model 5702 with rotor A-4-38 (Eppendorf SE; Hamburg, Germany), the patients’ plasma was split into 5 tubes and used for EVs isolation and in vitro experiments. The samples were stored at −80 °C at the Physiology laboratory of the Università del Piemonte Orientale (UPO), Novara, and were always handled in pseudonymized conditions.

**Figure 9:**
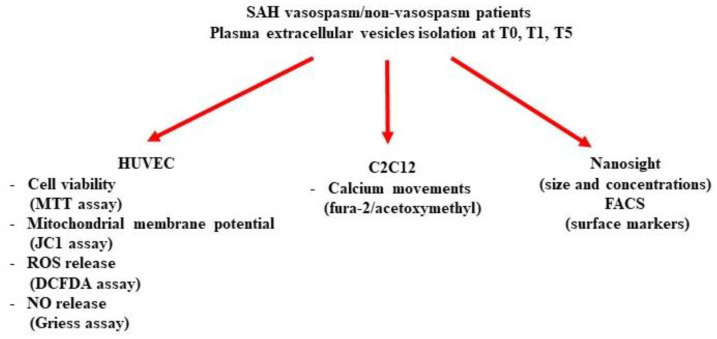
Flowchart of the experimental protocol.

In Figure 9, the experimental protocol is summarized.

### 4.3. EVs Isolation

EVs were isolated by ultracentrifugation (Beckman Coulter Optima™ LE-80K; Indianapolis, IN, USA). Briefly, 2 mL of plasma sample was diluted in test tubes with phosphate buffer saline (PBS) until reaching the final volume of 4 mL (Beckman Coulter, Milan, Italy).

Each blood sample was centrifuged for 20 min at 6000 *g* to remove cells, platelets, apoptotic bodies, and other large particles and aggregates, as previously performed [64]. Aliquots of plasma were maintained at −80 °C until use or further centrifuged to isolate extracellular vesicles. The enrichment of EVs was performed through ultracentrifugation at 100,000× *g* at 4 °C for 2 h with the SW60Ti rotor in a Beckman Coulter Optima L-90 K ultracentrifuge (Beckman Coulter, Fullerton, CA, USA). After the removal of the supernatant, the pellet was re-suspended in Dulbecco’s Modified Eagle Medium (DMEM) supplemented with 1% dimethyl sulfoxide (DMSO) and stored at −80 °C, as previously described [64,65,66].

### 4.4. EVs Characterization

The isolated EVs were diluted 1:200 in a 0.1 µm filtered physiological solution (NaCl 0.9%; Euroclone, Pero, Italy) and analyzed by NanoSight (NS300; Malvern Panalytical; Malvern, UK) equipped with a Nanoparticle Tracking Analysis (NTA) and NTA 3.2 Analytical Software Update). A syringe pump flow rate of 30 was applied for each sample. Three videos of 60 s each were recorded and analyzed, calculating an average number of EV size and concentration (particles/mL). Furthermore, fluorescence-activated cell sorting (FACS) was used to examine the expression of CD81 (EVs exosomal marker), CD3, CD4, CD8, CD154 and CD20 (EVs surface lymphocytes markers), CD41a, CD41b, CD42b (EVs surface platelets markers) and CD62p, CD62e (E selectin), CD105 and CD146 (EV surface endothelial markers) and CD142 (EVs surface tissue factor, TF). FACS analysis was performed by means of an Attune™ NxT flow cytometer (Thermo Fisher Scientific; Waltham, MA, USA), as previously performed [67].

Prior to FACS measurements, the EVs were diluted 1:100 with PBS in 1.5 mL tubes, which was followed by the addition of a fluorescently-labeled antibody specific to the surface markers of interest added to the EVs in a 1:1 ratio. After the addition of the antibody, the plate was incubated 1 h at 4 °C protected from light. The EVs isolated from the plasma of SAH patients and HC were plated and analyzed in triplicate or more. Comparisons were made vs. untreated cells. FITC-conjugated antibodies (BD Biosciences; San Jose, CA, USA) were used for CD41a, CD8, CD154, CD20, CD41b, CD42b, CD62p, and CD142, whereas PE-conjugated antibodies (BD Biosciences) were used for CD4, CD3, CD81, CD62e, CD105, and CD146. As a control, EVs from SAH patients were also stained with FITC and PE mouse Isotypic IgG (BD Biosciences).

### 4.5. Human Umbilical Vascular Endothelial Cells (HUVECs) and Smooth Muscle Cells (SMCs)

HUVECs and SMCs (C2C12) were purchased from ATCC (Manassas, VA, USA) (catalog No. CRL-1730 and CRL-1772, respectively) and maintained in DMEM (Euroclone) containing 2 mM L-glutamine (Euroclone), 1500 mg/L sodium bicarbonate (Euroclone) supplemented with 0.1 mg/mL heparin (Merck, Milan, Italy), 1% penicillin, 1% streptomycin, and 10% FBS (Euroclone). To examine the effects of the EVs from SAH patients vs. HC, HUVECs cultured in 96-well plates at a density of 5 × 10^3^ cells/well were stimulated with 50,000 EVs, diluted in PBS, per cell. The following assays were performed: cell viability (MTT assay), mitochondrial membrane potential, JC1 assay), ROS release (DCFDA assay), and NO release (Griess assay). In the initial part of the study, a time-course series of experiments was conducted to analyze the effects of EVs taken at T0 and administered to HUVECs for 12 h, 24 h, and 48 h on cell viability, mitochondrial membrane potential, ROS release, and NO release. Subsequently, a specific time point (T1 or T5) was chosen for all other experiments on HUVEC and C2C12 cells. Untreated HUVECs (cells stimulated with PBS only) were used as the negative control for comparison.

For C2C12 cells cultured in 96-well plates at a density of 5 × 10^3^ cells/well, the effects of EVs (50,000 EVs/cell) on calcium movements were evaluated using Fura-2/acetoxymethyl (AM) ester.

The experiments were performed in triplicate and repeated at least three times by using different pools of HUVEC and C2C12 cells.

### 4.6. MTT Assay

The cell viability of HUVECs was investigated by a 1% 3-[4,5-dimethylthiazol-2-yl]-2,5-diphenyl tetrazolium bromide (MTT) assay (Cayman Chemical, Ann Arbor, MI, USA), following established protocols in similar cellular models [66,67,68,69,70,71]. A 10% MTT solution was prepared by dissolving 50 mg of the MTT reagent (3-(4,5-dimethylthiazol-2-yl)-2,5-diphenyl tetrazolium bromide) in 10 mL of PBS (pH 7.4) and stored at 4 °C protected from light. After stimulating HUVECs with EVs for 24 h at the designated concentration, the media was removed, and 100 µL of the MTT solution, diluted in phenol-red-free DMEM high glucose supplemented with 2 mM L-glutamine and 1% penicillin–streptomycin (P/S), was added to each well. Thereafter, the plate was incubated at 37 °C for 2 h. Following incubation, the supernatant was discarded, and the formazan crystals formed in each well were dissolved with 100 µL of dimethyl sulfoxide (DMSO; Sigma, Milan, Italy). Cell viability was determined by measuring the absorbance through a spectrophotometer (VICTOR™ X Multilabel Plate Reader; PerkinElmer; Waltham, Massachusetts, USA) with a wavelength of 570 nm. Cell viability was calculated by setting the absorbance of control cells (untreated cells) at 100%.

### 4.7. JC-1 Assay

Mitochondrial membrane potential (ΔψM) was evaluated as an indicator of cell health, following established protocols in similar cellular models [66,67,68,69,70,71]. To assess the mitochondrial membrane potential, the medium of HUVECs stimulated with EVs (as described for the MTT assay) was removed, and cells were incubated with a 5,5′,6,6′-tetrachloro-1,1′,3,3′-tetraethylbenzimidazolyl carbocyanine iodide (JC-1) staining solution diluted in Assay Buffer 1X (Cayman Chemical; Ann Arbor, MI, USA) for 20 min at 37 °C, following the manufacturer’s instructions. After incubation, HUVECs were washed twice with assay buffer 1X, and then 100 µL/well of the buffer was added for the final reading. The mitochondrial membrane potential was determined by measuring the red (excitation 535 nm/emission 595 nm) and green (excitation 485 nm/emission 535nm) fluorescence using a spectrophotometer (VICTOR™ X Multilabel Plate Reader; PerkinElmer). The data were normalized to the fluorescence intensity of control cells (untreated cells).

### 4.8. DCFDA Assay

ROS generation in HUVECs was assessed by measuring the oxidation of 2,7-dichlorodihydrofluorescein diacetate (H2DCFDA) into 2,7-dichlorodihydrofluorescein (DCF), following the manufacturer’s instructions (Abcam; Cambridge, UK), and as previously performed [66,71,72]. After stimulating HUVECs with EVs, as described for MTT and JC assays, the medium was removed, and cells were stained with 10 μM H2DCFDA for 20 min at 37 °C. The fluorescence intensity of DCF was measured at an excitation and emission wavelength of 485 and 530 nm, respectively, using a spectrophotometer (VICTOR™ X Multilabel Plate Reader; PerkinElmer). The results were expressed as DCF fluorescence intensity, which is proportional to the amount of intracellular ROS. The data were normalized to the fluorescence intensity of control cells (untreated cells).

### 4.9. NO Release

NO release in HUVECs was quantified using the Griess method (Promega, Milan, Italy) [68,73]. Following the stimulation of HUVECs as described for previous assays, the supernatants were collected to measure NO production. An equal volume of Griess reagent was added to each sample according to the manufacturer’s instructions. The absorbance of each sample was read at 540 nm using a spectrometer (VICTOR™ X Multilabel Plate Reader). To quantify NO production, a standard curve was prepared, and the results were expressed as nitrites (μM).

### 4.10. Measurement of [Ca^2+^]_c_ by Fura-2 Fluorescence

To measure [Ca^2+^]_c_, C2C12 cells were grown to confluence, washed twice with sterile PBS 1× (Euroclone), and incubated with 5 μM fura-2/acetoxymethyl (AM) ester (Sigma, Milan, Italy) in DMEM (Euroclone) containing 10% FBS and without phenol red for 30 min in the dark. After additional washings with DMEM (Sigma), the coverslips were mounted in a thermostatted quartz cuvette and placed in an agitation system at 37 °C. The measurement was performed using a Hitachi F-4500 Fluorescence Spectrometer (Hitachi High-Technologies Corporation) for a continuous duration of 300 s at an excitation wavelength of 340 nm and an emission wavelength of 510 nm. Fura-2/AM-loaded C2C12 cells were stimulated with EVs as described for HUVECs, in the presence or absence of Ca^2+^ in the incubation medium with 50 mM ethylene glycol tetraacetic acid (EGTA) (Sigma). The quantification of [Ca^2+^]_c_ was obtained using the following equation, as previously reported [74,75,76]: (Ca^2+^) = *K*_d_ ((*R* − *R*_min_)/(*R*_max_ − *R*)). The *K*_d_ of fura-2/AM for Ca^2+^ was considered as 224. *R*_min_ and *R*_max_ were the minimum and maximum values of fluorescence ratio obtained under Ca^2+^-free (EGTA 0.1 M) or Ca^2+^-saturated conditions, respectively. The fluorescence intensities obtained were corrected for cell autofluorescence at the respective wavelengths employed [75].

### 4.11. Statistical Analysis

All data were collected and managed using the Research Electronic Data Capture software (RED-Cap, Vanderbilt University, Nashville, TN, USA). Clinical data for quantitative variables are presented as the median and interquartile range (IQR). In the case of data from in vitro experiments, the results are presented as the mean ± standard deviation (SD) of repeated measurements. The differences between two groups were assessed using the Mann–Whitney test. A value of *p* < 0.05 was considered statistically significant. All statistical analyses were performed using Graph PAD 6.0 (GraphPad Software, San Diego, CA, USA).

## 5. Conclusions

In summary, the results of this study uncover the existence of an altered pattern of circulating EVs in SAH patients, detectable as early as 24 h post-SAH. Significant differences were also found between VP and NVP patients in circulating EVs after seven days. Furthermore, our data demonstrate the pathogenic role of EVs in inducing endothelial damage, characterized by loss of mitochondrial function, increased oxidative stress, and intracellular calcium elevation in SMCs, especially in VP patients. The information collected in vitro could be useful to implement knowledge about EVs in the pathophysiology of SAH, since they support the hypothesis that EVs could play a key role in the onset of both VP and DCI. Also, our data highlight the potential of the use of EVs as diagnostic/prognostic markers, as well as therapeutic tools. The current diagnostic and prognostic tools for SAH are often invasive and may have limitations. However, the characterization of EVs offers a promising non-invasive approach for screening SAH patients and has the potential to contribute to improved prognoses [30].

Overall, the analysis of EVs cargo may provide valuable insights into the pathophysiology of VP and serve as a potential biomarker for early detection and risk stratification.

Further research and validation are, however, necessary to harness the full potential of EVs characterization in SAH management and to translate these findings into clinical practice.

In particular, in order to achieve those objectives, it will be mandatory to increase the sample size and better characterize the EVs population. The limitations of the present study are, in fact, related to the low number of patients enrolled, which is secondary to the Center’s recruitment capacity, and to the lack of a detailed EVs analysis according to MISEV, MIFlowCyt, and MIFlowCyt-EV guidelines [77].

## Figures and Tables

**Table 1:** Patient and healthy control demographic and clinical characteristics.

Demographic and Clinical Characteristics	All Patients *n* = 18	Healthy Controls *n* = 5	*p* Value
Age (y)	54.5 (71–47.5)	53 (46.5–61.5)	0.5
Sex			>0.99
Male (%)	8 (44)	2 (40)	
Female (%)	10 (56)	3 (60)	
Comorbidities			>0.99
Hypertension (%)	6 (33)	1 (20)	
Cardiovascular disease (%)	2 (11)	0 (0)	
Respiratory disease (%)	1 (5)	0 (0)	
Chronic kidney disease (%)	1 (5)	0 (0)	
Cancer (%)	1 (5)	0 (0)	
Other (%)	4 (22)	1 (20)	

Data are presented as a number and percentage or median and IQR.

**Table 2:** Patient demographic and clinical characteristics.

Demographic and Clinical Characteristics	All Patients *n* = 18	Vasospasm Patients *n* = 8	Non-Vasospasm Patients *n* = 10	*p* Value
Age (y)	54.5 (71–47.5)	49.5 (52.8–43.5) *	70.5 (73.5–57.8) *	0.003
Sex				0.19
Male (%)	8 (44)	2 (25)	6 (60)	
Female (%)	10 (56)	6 (75)	4 (40)	
Aneurysm site				0.08
MCA (%)	4 (22)	3 (37.5)	1 (10)	
ACA (%)	6 (33)	4 (50)	2 (20)	
BA (%)	3 (17)	1 (12.5)	2 (20)	
Other (%)	5 (28)	0 (0)	5 (50)	
Aneurysm treatment				0.61
Surgical clipping (%)	5 (28)	3 (38)	2 (20)	
Endovascular coiling (%)	13 (72)	5 (62)	8 (80)	
GCS at ICU admission	7 (9–6)	8 (9–4)	7 (10–6)	0.71
WFNS scale at ICU admission	4.5 (5–4)	4 (5–4)	5 (5–3.5)	0.63
Fisher scale at ICU admission	3.5 (4–3)	3 (3–3)	4 (4–3.8)	0.06
HHS at ICU admission	4 (5–3)	4 (5–3)	4.5 (5–3)	0.64
ICU LOS (d)	21 (33.5–11)	18.5 (34.5–11)	22 (33–12.3)	0.88
Hospital LOS (d)	29 (46.3–14)	19 (42.5–11)	36.5 (50–12.3)	0.25
mRS				>0.99
3 months	6 (6–4)	6 (6–5.3)	5 (6–3.5)	
6 months	6 (6–4)	6 (6–4.5)	5 (6–3.5)	
GOS-E				>0.99
3 months	1 (3–1)	1 (2.5–1)	3 (3–1)	
6 months	1 (3–1)	1 (2.5–1)	3 (3–1)	

Data are presented as a number and percentage or median and IQR. * *p* < 0.05. MCA, middle cerebral artery; ACA, anterior cerebral artery; BA, basilar artery; GCS, Glasgow coma score; ICU, intensive care unit; WFNS, World Federation of Neurosurgical Societies scale; HHS, Hunt and Hess grading system; LOS, length of stay; mRS, modified Rankin scale; GOS-E, Glasgow outcome scale—extended.

**Table 3:** Percentage of each type of circulating EV among the total EV populations.

	T0		T1		T5	
	NVP	VP	NVP	VP	NVP	VP
CD4	5.8	6	4.2	4.9	3	5
CD8	5	5.6	5.8	6.5	3	6
CD42b	14	15	13.4	16.4	11	13.5
CD142	11	13	8.3	14.5	9	11
CD20	10.7	11	10.5	11.3	10	11.3
CD41a	4,7	7	6.5	7	4	5.7
CD41b	8	11	8.4	9.6	5	5.9
CD3	9.5	9.5	7.7	6.8	8.5	6.6
CD154	5.3	4.8	6,1	6.3	4.6	6.5
CD62e	14	5	11.1	4	17	7.3
CD62p	8.7	10.4	9.2	11.5	8.9	15
CD146	2.3	1.2	3.8	0.2	6	2
CD105	1	0.5	5	1	10	4.2

NVP: non-vasospasm patients; VP: vasospasm patients.

**Table 4:** Effects of EVs on intracellular calcium in C2C12 cells in the presence or absence of EGTA.

		T0			T1			T5		*p*
	Basal	EGTA	EVs	Basal	EGTA	EVs	Basal	EGTA	EVs	
VP	100 ± 3.4	91.5 ± 2.4 *	99.4 ± 3.1 *	101 ± 2.9	93 ± 3.2 *	100.8 ± 2.7 *	100.4 ± 2.7	92.2 ± 3.2 *	93.3 ± 3.3 *	* <0.05
NVP	99.4 ± 2	91.2 ± 2.3 *	95.5 ± 1.6 *	100 ± 2.1	91.7 ± 1.8 *	94.8 ± 1.7 *	100.8 ± 2.7	92.4 ± 3.1 *	92.6 ± 3 *	* <0.05

The results are expressed as the means ± SD of experiments performed in triplicate and repeated at least three times. Measurements are expressed in nM. Basal: intracellular calcium before EGTA. EVs: extracellular vesicles. EGTA: ethylene glycol tetraacetic acid. VP: vasospasm patients. NVP: non-vasospasm patients. * *p <* 0.05 vs. basal.

## Data Availability

Data are available on reasonable request.
